# High-coverage sequencing and annotated assembly of the genome of the Australian dragon lizard *Pogona vitticeps*

**DOI:** 10.1186/s13742-015-0085-2

**Published:** 2015-09-28

**Authors:** Arthur Georges, Qiye Li, Jinmin Lian, Denis O’Meally, Janine Deakin, Zongji Wang, Pei Zhang, Matthew Fujita, Hardip R. Patel, Clare E. Holleley, Yang Zhou, Xiuwen Zhang, Kazumi Matsubara, Paul Waters, Jennifer A. Marshall Graves, Stephen D. Sarre, Guojie Zhang

**Affiliations:** 1 Institute for Applied Ecology, University of Canberra, Canberra, ACT 2601 Australia; 2 China National GeneBank, BGI-Shenzhen, Shenzhen, 518083 China; 3 Centre for GeoGenetics, Natural History Museum of Denmark, University of Copenhagen, Øster Voldgade 5-7, Copenhagen, 1350 Denmark; 4 School of Bioscience and Bioengineering, South China University of Technology, Guangzhou, 510006 China; 5 School of Biotechnology & Biomolecular Sciences, University of New South Wales, Sydney, NSW 2052 Australia; 6 John Curtin School of Medical Research, Australian National University, Canberra, ACT 2601 Australia; 7 Department of Biology, University of Texas at Arlington, 701 S. Nedderman Drive, Arlington, TX 76019 USA; 8 School of Life Science, La Trobe University, Melbourne, VIC 3086 Australia; 9 Centre for Social Evolution, Department of Biology, University of Copenhagen, Universitetsparken 15, DK-2100 Copenhagen, Denmark

**Keywords:** *Pogona vitticeps*, Dragon lizard, Central bearded dragon, Agamidae, Squamata, Next-generation sequencing

## Abstract

**Background:**

The lizards of the family Agamidae are one of the most prominent elements of the Australian reptile fauna. Here, we present a genomic resource built on the basis of a wild-caught male ZZ central bearded dragon *Pogona vitticeps*.

**Findings:**

The genomic sequence for *P. vitticeps*, generated on the Illumina HiSeq 2000 platform, comprised 317 Gbp (179X raw read depth) from 13 insert libraries ranging from 250 bp to 40 kbp. After filtering for low-quality and duplicated reads, 146 Gbp of data (83X) was available for assembly. Exceptionally high levels of heterozygosity (0.85 % of single nucleotide polymorphisms plus sequence insertions or deletions) complicated assembly; nevertheless, 96.4 % of reads mapped back to the assembled scaffolds, indicating that the assembly included most of the sequenced genome. Length of the assembly was 1.8 Gbp in 545,310 scaffolds (69,852 longer than 300 bp), the longest being 14.68 Mbp. N50 was 2.29 Mbp. Genes were annotated on the basis of *de novo* prediction, similarity to the green anole *Anolis carolinensis*, *Gallus gallus* and *Homo sapiens* proteins, and *P. vitticeps* transcriptome sequence assemblies, to yield 19,406 protein-coding genes in the assembly, 63 % of which had intact open reading frames. Our assembly captured 99 % (246 of 248) of core CEGMA genes, with 93 % (231) being complete.

**Conclusions:**

The quality of the *P. vitticeps* assembly is comparable or superior to that of other published squamate genomes, and the annotated *P. vitticeps* genome can be accessed through a genome browser available at https://genomics.canberra.edu.au.

**Electronic supplementary material:**

The online version of this article (doi:10.1186/s13742-015-0085-2) contains supplementary material, which is available to authorized users.

## Data description

The central bearded dragon, *Pogona vitticeps*, is widespread through the arid and semi-arid regions of eastern central Australia. This lizard adapts readily to captivity, lays large clutches of eggs several times per season, and is kept as a favoured pet species in Europe, Asia and North America. The karyotype of *P. vitticeps* is typical of most Australian agamids, consisting of six pairs of macrochromosomes and ten pairs of microchromosomes (2n = 32) [1]. The sex determining mechanism is one of female heterogamety (ZZ/ZW) and the sex chromosomes are a pair of microchromosomes [2]. Sex determination, a primary driver for our interest in generating this genome sequence, is complex in this species, involving an interaction between the influences of incubation environment and the ZZ/ZW genotype [3, 4].

### Samples and sequencing

DNA samples were obtained from a blood sample taken from a single male *Pogona vitticeps* (Fabian, UCID 001003387339) verified as a ZZ male using sex-linked polymerase chain reaction (PCR) markers [3] and cytological examination [2]. This work was undertaken in accordance with the Australian Capital Territory Animal Welfare Act 1992 and the approval of the University of Canberra Animal Ethics Committee. DNA was extracted and purified using standard protocols and transported to BGI-Shenzhen, China for sequencing. 13 insert libraries were constructed with insert sizes of 250 bp, 500 bp, 800 bp, 2 kbp (*x*2), 5 kbp, 6 kbp, 10 kbp (*x*2), 20 kbp (*x*2) and 40 kbp (*x*2) and subjected to paired-end sequencing on an Illumina HiSeq 2000 platform to generate 317 Gbp of raw sequence (Table 1). After filtering for low-quality reads and duplicated reads arising from PCR amplification during library construction, 146.38 Gbp of data were retained for genome assembly. This amount of data represents an average read depth of 82.7 (Table 1), assuming a genome size of 1.81 pg, as estimated for a female *P. vitticeps* by flow cytometry [5]. This mass converts to a genome size of 1.77 Gbp [6].

**Table 1:** Summary of sequencing data derived from paired-end sequencing of 13 insert libraries using an Illumina HiSeq 2000 platform

			Raw data				Filtered data			
Insert size (bp)	Accession numbers	Nunber of libraries	Read length (bp)	Raw data (Gbp)	Average read depth (X)	Physical coverage (X)	Read length (bp)	Filtered data (Gbp)	Average read depth (X)	Physical coverage (X)
250	ERR409943	1	150	55.17	31.17	25.97	125	42.49	24.01	24.00
	ERR409944									
500	ERR409945	1	150	34.32	19.39	32.32	125	23.66	13.37	26.72
	ERR409946									
800	ERR409947	1	150	46.28	26.15	69.72	125	32.2	18.19	60.63
2,000	ERR440173	2	49	38.39	21.69	442.64	49	18.19	10.28	209.73
	ERR409948									
5,000	ERR409949	1	49	17.48	9.88	503.95	49	6.56	3.71	188.99
6,000	ERR409950	1	49	17.43	9.85	603.01	49	6.01	3.4	208.00
10,000	ERR409951	2	49	34.94	19.74	2,014.60	49	7.89	4.46	455.00
	ERR409952									
20,000	ERR409953	2	49	38.53	21.77	4,443.48	49	6.63	3.75	764.38
	ERR409954									
40,000	ERR409955	2	49	34.4	19.44	7,932.30	49	2.75	1.55	633.29
	ERR409956									
		13		316.94	179.06	16,067.99		146.38	82.7	2,570.74

Read depth was calculated on the basis of a genome size of 1.77 Gbp. Average read depth, number of times on average a particular base is included in a read. Physical coverage, the number of times on average a particular base is spanned by a paired read

**Fig. 1:**
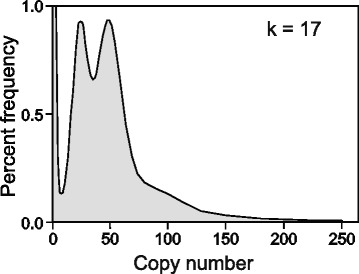
K-mer spectrum for the genome sequence of a male *Pogona vitticeps* (ZZ). Sequencing DNA derived from the short-insert libraries (250, 500, 800 bp) yielded 98.35 Gbases of clean data in the form of 125 bp reads, which generated 76.89x109 17-mer sequences. The solid line shows the k-mer spectrum (percentage frequency against k-mer copy number). The second mode (copy number 48.5) represents homozygous single copy sequence, whereas the first mode (24.5), half the copy number of the first, represents heterozygous single copy sequence. Heterozygosity is high, which complicated assembly

Reads from the short-insert libraries (250, 500 and 800 bp) were decomposed into short sequences of length k (k-mers, with *k* = 17) using Jellyfish version 1.1 [7]. The histogram of k-mer copy number (Fig. 1) was strongly bi-modal, the first mode with a copy number that was half that of the second, which reflects the high level of heterozygosity in this wild-caught lizard (0.85 % of single nucleotide polymorphisms [SNPs] plus sequence insertions or deletions [indels]). The second mode in the k-mer graph was used to obtain an estimate of the genome size using the formula:

$$Average\ \mathrm{R}\mathrm{e} ad\ Depth = Mode.\frac{L}{L-k+1}=55.62\ \mathrm{fold}$$

$$Genome\ Size = \frac{Total\ of\ \mathrm{R}\mathrm{e} ad\ Lengths}{Average\ \mathrm{R}\mathrm{e} ad\ Depth}=1.768\ Gbp$$

where L is the read length (125 bp), k is the k-mer length (17 bp), there are 98.35 Gbp of sequence data, and the mode is taken from the k-mer graph (48.5, Fig. 1). Our sequence-based estimate of 1.768 Gbp agrees well with the estimate of 1.77 Gbp for the ZW genome that was previously made using flow cytometry data [4].

### Assembly

Assembly was performed with SOAPdenovo (version 2.03) [8, 9]. Briefly, the sequences derived from the short-insert libraries were decomposed into k-mers to construct the *de Bruijn* graph, which was simplified to allow connection of the remaining k-mers into contiguous sequence (contigs). We then aligned the paired-end reads from small and large-insert library sets to the contigs, calculated the support for relationships between contigs, assessed the consistent and conflicting relationships, and constructed scaffolds. Finally, we retrieved paired reads that mapped to a unique contig but had the other member of the pair located in a gap region. Reads falling in the same gap region were then assembled locally. The final assembly (European Nucleotide Archive [ENA] accession number ERZ094017) yielded a contig N50 of 31.3 kbp and a scaffold N50 of 2.3 Mbp (N50 meaning that 50 % of the genome sequence is contained in contigs, or scaffolds, equal to or greater than this length), with unclosed gap regions representing only 3.78 % of the assembly (Table 2).

**Table 2:** Statistics for the assembly contigs and scaffolds (after gap filling)

	Contig		Scaffold	
	Size (bp)	Number	Size (bp)	Number
N90	4,850	63,958	200,992	1,095
N80	12,159	42,491	670,865	644
N70	18,332	30,884	1,149,567	441
N60	24,540	22,654	1,671,674	311
N50	31,298	16,344	2,290,546	219
Longest	295,776		14,681,335	
Total size	1,747,524,961		1,816,115,349	
≥100 bp		636,524		545,300
≥2 kbp		79,002		4,356
Gap ratio		0 %		3.78 %

Reads from small-insert libraries that satisfied our filtering criteria were aligned to the assembly using the Burrows-Wheeler Aligner (BWA, version 0.5.9-R16) [10], allowing for eight mismatches and one indel. Of the total number of reads (797.4 M), 96.4 % could be mapped back to the assembled genome and they covered 98.4 % of the assembly excluding gaps. Bases in the assembled scaffolds had, on average, reads mapped with 55X read depth. These data suggest that we have assembled most of the *P. vitticeps* genome. In addition, we used the CEGMA package (version 2.4) [11] to map 248 core eukaryotic genes to our *P. vitticeps* assembly. Our assembly captured 99 % (246 of 248) of the core CEGMA genes, with 93 % (231) being complete. This is a higher assembly rate than that estimated for the green anole *Anolis carolinensis* assembly (AnoCar2.0), which captured 93.6 % (232) of the core genes, with 85.9 % (213) being complete.

### Transcriptomes

We generated transcriptome data from the brain, heart, lung, liver, kidney, skeletal muscle and gonads of male and female *P. vitticeps* (Table 3). None of the seven animals from which we collected RNA was used in generating the genome sequence. Two sets of sequencing runs on two different male and female individuals were performed by BGI-Shenzen, producing 309,436,077 90 bp paired-end reads (ENA accession numbers ERR753524-ERR753530 and ERR413064-ERR413076). A third set of samples was sequenced by The Ramaciotti Centre, University of New South Wales, Australia, including a sex-reversed ZZ female, producing 89,687,526 101 bp paired-end reads (ENA accession numbers ERR413077- ERR413082). We assembled these datasets (from all seven individuals) into 595,564 contigs using Trinity (release r2013_08_14) [12] with default parameters (ENA accession number ERZ097159). Only the first set of RNA-seq reads was available for genome annotation (ENA accession numbers ERR753524-ERR753530) but we make the entire dataset, including our *de novo* assembly, available with this article (see ‘Availability of supporting data’ section).

**Table 3:** Number of predicted genes with RNA-seq signals

Specimen ID (tissue ID)	Accession number	Tissue	Genotype	Phenotype	RPKM >0		RPKM >1		RPKM >5	
					Number	Ratio (%)	Number	Ratio (%)	Number	Ratio (%)
1003347859	ERR753524	Brain	ZZ	Intersex	17,049	87.85	14,403	74.22	11,244	57.94
(AA45100)										
1003338787	ERR753525	Brain	ZZ	Male	16,934	87.26	14,467	74.55	11,359	58.53
(AA60463)										
1003348364	ERR753526	Brain	ZW	Female	17,121	88.23	14,526	74.85	11,474	59.13
(AA60435)										
1003347859	ERR753527	Testes	ZZ	Intersex	16,874	86.95	13,874	71.49	10,784	55.57
(AA45100)										
1003347859	ERR753528	Ovary	ZZ	Intersex	16,827	86.71	12,952	66.74	10,421	53.7
(AA45100)										
1003338787	ERR753529	Testes	ZZ	Male	17,963	92.56	14,951	77.04	11,311	58.29
(AA60463)										
1003348364	ERR753530	Ovary	ZW	Female	17,188	88.57	13,634	70.26	10,946	56.41
(AA60435)										
Combined					18,833	97.05	17,646	90.93	15,974	82.31

Gene expression levels were measured as RPKM (reads per kilobase of gene per million mapped reads). Ratios are based on a total of 19,406 annotated protein-coding genes

### Annotation

Transposable elements and other repetitive elements were identified using a combination of homology, at both the DNA and protein levels, and *de novo* prediction. In the homology-based approach, we searched Repbase [13] for known transposable elements, used RepeatMasker [14] for DNA homology search against the Repbase database, and used WuBlastX to search against the transposable element protein database provided within RepeatProteinMask (bundled in RepeatMasker). In the *de novo* approach, we used RepeatModeler [15] and LTR_FINDER [16] to predict repetitive elements. Tandem repeats were identified using Tandem Repeats Finder [17]. The relative success of the different approaches is shown in Table 4. Overall, we identified about 690 Mbp of repetitive sequences accounting for 39.47 % of the genome, of which the predominant elements were long interspersed nuclear elements (LINEs, which accounted for 33 % of repetitive sequences representing 12.2 % of the genome) (Table 5).

**Table 4:** The statistics for repeats in the *P. vitticeps* genome annotated by different methods

Program	Total repeat length (bp)	Percentage of genome
Tandem Repeats Finder	59,773,950	3.42
Repeatmasker	174,011,206	9.96
Proteinmask	157,050,977	8.99
RepeatModeler	592,771,829	33.92
LTR Finder	65,464,996	3.75
Total	689,687,572	39.47

**Table 5:** Breakdown of repeat content of the *Pogona vitticeps* genome derived from RepeatMasker analysis

Category	Repbase TEs		TE proteins		*de novo*		Combined TEs	
	Length (bp)	% of genome	Length (bp)	% of genome	Length (bp)	% of genome	Length (bp)	% of genome
DNA	25,035,683	1.43	6,450,126	0.37	56,943,252	3.26	70,663,766	4.04
LINE	124,676,466	7.13	132,747,210	7.60	191,015,014	10.93	213,508,152	12.22
SINE	20,281,741	1.16	-	0.00	54,941,907	3.14	57,180,364	3.27
LTR	7,613,766	0.44	17,931,338	1.03	16,104,019	0.92	28,021,391	1.60
Other	24,327	0.00	-	0.00	-	0.00	24,327	0.00
Unknown	761,119	0.04	-	0.00	283,563,847	16.23	284,276,315	16.27
Total	174,011,206	9.96	157,050,977	8.99	627,828,869	35.93	657,625,603	37.63

*Abbreviations*: *LINE* long interspersed nuclear element, *LTR* long terminal repeat, *SINE* short interspersed nuclear element, *TE* transposable element

We combined homology-based, *de novo* and transcriptome-based methods to predict gene content of the assembly. In the homology-based prediction, the assembly was annotated by generating reference sets of *A. carolinensis*, *Gallus gallus* and *Homo sapiens* proteins, and aligning the reference sets to the assembly using TBLASTN (version 2.2.23; E-value ≤ 1 × 10^−5^). The resultant homologous genome sequences were then aligned against matching proteins using Genewise (version wise2-2-0) [18] to define gene models. In the *de novo* prediction, we randomly selected 1,000 genes with intact open reading frames (ORFs) as predicted by the homology-based approach to train the Augustus gene prediction tool (version 2.5.5) [19] with the parameters appropriate to *P. vitticeps*. The *de novo* gene prediction was then performed with Augustus applied to the genome after repeat sequences were masked as described above. In the transcriptome-based approach, we mapped transcriptome reads to the assembly using TopHat (version 1.3.1) [20], which can align reads across splice junctions. These mapped reads were assembled into transcripts using Cufflinks (version 1.3.0) [21] and then merged across samples (*n* = 7, Table 3) into a single transcriptome annotation using the Cuffmerge option.

The results of the three approaches were combined into a non-redundant gene set of 19,406 protein-encoding genes, 63 % of which included intact ORFs (Table 6). Most of the predicted genes were supported by RNA-seq signals (Table 3).

**Table 6:** Characteristics of predicted protein-coding genes in the *Pogona vitticeps* assembly and comparison with *Anolis carolensis*, *Gallus gallus* and *Homo sapiens*

Gene set		Total	Intact ORF	Single exon gene	Gene length (bp)	mRNA length (bp)	Exons per gene	Exon length (bp)	Intron length (bp)
Homolog	*Anolis carolinensis*	16,009	2,583	1,668	23,021	1,524	8.57	178	2,839
	*Gallus gallus*	12,727	2,068	1,509	27,608	1,558	9.06	172	3,232
	*Homo sapiens*	13,544	2,456	1,250	32,551	1,699	9.75	174	3,528
	Combined	18,033	3,263	2,180	26,631	1,577	8.93	177	3,160
*De novo* (Augustus)		32,110	32,110	6,767	14,109	1,125	6.07	185	2,561
Transcriptome		22,986	14,555	2,951	12,511	1,214	6.99	174	1,885
Merged		19,406	12,172	1,999	26,215	1,642	9.24	178	2,984
Other species	*Anolis carolinensis*	17,805	4,280	1,372	23,469	1,526	9.55	160	2,566
	*Gallus gallus*	16,736	7,777	1,684	21,314	1,438	9.35	154	2,379
	*Homo sapiens*	21,849	20,905	2,602	46,301	1,635	9.44	173	5,293

Except for the columns headed Total, Intact ORF and Single exon gene, the values presented are means.

*Abbreviation*: *ORF* open reading frame

To assign gene names to each predicted protein-coding locus, we mapped the 19,406 genes to an Ensembl library collated from *A. carolinensis*, chicken *G. gallus*, human *H. sapiens*, western clawed frog *Xenopus tropicalis* and zebrafish *Danio rerio.* The name associated with the best hit for each *P. vitticeps* gene was assigned to each of 19,083 genes. Most of these genes (16,510) mapped to a homolog even at high stringency (>80 % of protein length aligned).

### Bacterial artificial chromosome library

A large-insert genomic DNA bacterial artificial chromosome (BAC) library was constructed from DNA from a wild-caught female dragon lizard (TC1542) confirmed to have the ZW genotype using sex-linked PCR markers [3, 4] and cytologically [3]. The library is estimated to represent 6.3× of genome coverage, and is comprised of 92,160 clones with an average insert size of 120 kbp. This resource is commercially available through Amplicon Express (Pullman, WA, USA; http://ampliconexpress.com).

### Anchoring sequences to chromosomes

Our previously published cytogenetic map of *P. vitticeps* consisted of 87 BACs that were mapped to the macrochromosomes (64 BACs) and microchromosomes (23 BACs) [1]. We mapped an additional 80 BACs, extending the set to 125 markers on macrochromosomes and 42 on microchromosomes. Sequence scaffolds were anchored to chromosomes by 52 loci, contained in the BACs, that are conserved in homologous syntenic blocks across amniotes (*A. carolinensis*, *G. gallus*, *H. sapiens*). By using gene synteny information 37.9 % (670 Mbp) of the sequenced genome has been assigned to chromosomes (Deakin et al., unpublished data).

### Sex chromosome sequences

The sex of *P. vitticeps* is determined by a combination of chromosomal constitution and influence of environmental temperature on the developing embryo. *P. vitticeps* has female heterogamety (with ZZ male and ZW female individuals), and the Z and W chromosomes are among the ten pairs of microchromosomes [2]. Sex chromosome heteromorphy is evident by C-banding, but the degree of differentiation of the Z and W chromosomes is slight [2]. The sex chromosomes of *P. vitticeps* are not homologous to the sex chromosomes of chicken (*G. gallus*) or other reptiles so far examined [22]. The ZZ genotype is reversed to a female phenotype at high incubation temperatures [3, 4].

Our laboratory has previously identified a sex-linked sequence using amplified fragment length polymorphism screening and genome walking [4, 23]. Five contiguous BAC clones containing sex-linked markers that map to the sex chromosome pair were sequenced to reveal 352 kbp of *P. vitticeps* sex chromosome sequence [24]. This region contained five protein-coding genes (*oprd1*, *rcc1*, *znf91*, *znf131* and *znf180*) and several major families of repetitive sequences (long terminal repeat [LTR] and non-LTR retrotransposons, including chicken repeat 1 [CR1] and bovine B LINEs [Bov-B LINEs]) [1, 24].

More recently, we amplified micro-dissected W-chromosome fragments to yield many sex chromosome sequence tags that were reciprocally mapped to their Z homologs (Matsubara et al., unpublished data). All putative sex chromosome scaffolds were confirmed to co-localize with the known ZW-BAC Pv3-L07 when physically mapped (Deakin et al., unpublished data). In this way we identified 12.8 Mbp of the Z chromosome (on three scaffolds) and increased the number of confirmed sex chromosome genes to 240 (Deakin et al., unpublished data).

### GC content and isochore structure

**Fig. 2:**
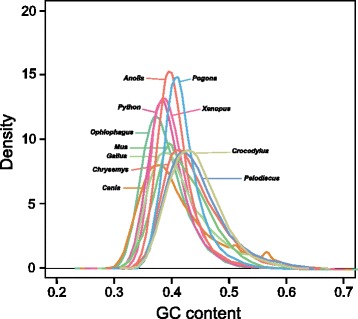
Distribution of GC content in 5 Kbp windows for a range of vertebrates including *Pogona vitticeps*

**Fig. 3:**
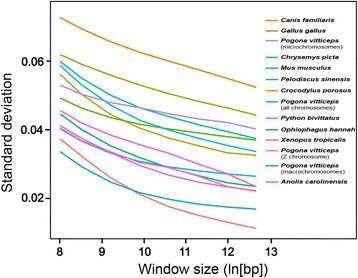
Variation in GC content among windows for various genome sequences with increasing window size (5, 10, 20, 40, 80, 160, and 320 Kb windows). The relationship for *Pogona vitticeps* is disaggregated to macrochromosomes, microchromosomes and the Z sex chromosome for comparison. Scale of X axis is natural logarithm. *Pogona* macrochromosomes share the lack of isochore structure reported for the *Anolis* genomeᅟ

**Fig. 4:**
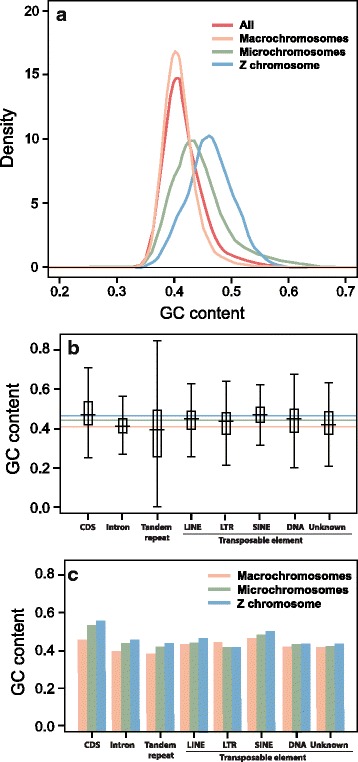
Analysis of GC content in *Pogona vitticeps*. **a**, Distribution of GC content in all chromosomes, macrochromosomes, microchromosomes and the Z chromosome, calculated with a non-overlap 5-kb sliding windows ; **b**, GC content of various components of the genome, in comparison with the average GC content for macrochromosomes (red line), microchromosomes (green line) and the Z chromosome (blue line) ; **c**, GC content of the macrochromosomes, microchromosomes and Z chromosomes broken down for various components of the genome

We investigated patterns of GC content variation in the *P. vitticeps* genome using two approaches. First, we examined the absolute GC content in non-overlapping 5 kbp windows for several genomes (*P. vitticeps*, *A. carolinensis* [25], Burmese python *Python bivittatus* [26], king cobra *Ophiophagus hannah* [27]*,* western painted turtle *Chrysemys picta* [28],Chinese softshell turtle *Pelodiscus sinensis* [29], saltwater crocodile *Crocodylus porosus* [30] chicken *G. gallus*, mouse *Mus musculus*, domestic dog *Canis familiaris* [31] and western clawed frog *X. tropicalis* [32]; Table 7; Fig. 2). We then examined variation in GC composition for these same genomes at increasing spatial scales (5, 10, 20, 40, 80, 160 and 320 kbp windows; Fig. 3). We also looked at different subsets of the *P. vitticeps* genome, including macrochromosomes and microchromosomes, and the Z chromosome (Fig. 4a), by restricting the analysis to scaffolds that have been physically mapped (Deakin et al., unpublished data).

**Table 7:** Comparison of mean GC content for available tetrapod genomes

Organism	Genome version	Mean GC	SD
*Pogona vitticeps*	pvi1.1.Jan.2013	0.418	0.037
*P. vitticeps* - microchromosomes	pvi1.1.Jan.2013	0.445	0.050
*P. vitticeps* - macrochromosomes	pvi1.1.Jan.2013	0.409	0.029
*P. vitticeps* - Z chromosome	pvi1.1.Jan.2013	0.469	0.037
*Xenopus tropicalis*	JGI_4.2	0.398	0.038
*Anolis carolinensis*	AnoCar2	0.403	0.032
*Canis familiaris*	CanFam3.1	0.413	0.069
*Mus musculus*	GRCm38	0.417	0.046
*Gallus gallus*	Galgal4	0.416	0.059
*Crocodylus porosus*	croc_sub2	0.442	0.050
*Pelodiscus sinensis*	PelSin_1.0	0.441	0.053
*Chrysemys picta*	ChrPicBel3.0.1	0.437	0.055
*Python bivittatus*	python_5.0	0.396	0.042
*Ophiophagus hannah*	GCA_000516915.1 (NCBI)	0.386	0.040

*Abbreviation*: *SD* standard deviation

The macrochromosomes of *P. vitticeps* are largely devoid of variation in GC content at small (5 kbp) spatial scales. In fact, *P. vitticeps* macrochromosomes are more uniform in terms of GC distribution than is the *A. carolinensis* genome (standard deviation 0.029 versus 0.032 respectively; Table 7). With the exception of the Z microchromosome, *P. vitticeps* microchromosomes possess a heterogeneous distribution of GC-rich sequences over 5 kbp windows (Fig. 4a). In this regard, *P. vitticeps* microchromosomes resemble those of birds but differ markedly from those of *A. carolinensis*, whose GC content more closely resembles that of the macrochromosomes [33]. Intriguingly, the Z microchromosome of *P. vitticeps* has an average GC content comparable to that of coding sequences and short interspersed nuclear elements (SINEs) (Fig. 4b), which suggests that this chromosome may be enriched in these GC-rich components of the genome. However, with the exception of LTR transposable elements, all components (CDS, introns, tandem repeats and transposable elements) showed greater GC content if they resided on the Z chromosome than elsewhere (Z chromosome > microchromosomes > macrochromosomes; Fig. 4c), suggesting that there are other, as yet unidentified, reasons for the observed variation in GC content across different chromosome classes.

When variation in GC distribution is considered over larger spatial scales (tens to hundreds of kbp, Fig. 3), the *P. vitticeps* macrochromosomes are similar to the *A. carolinensis* genome, which lacks substantial variation in GC composition, a striking departure from isochore patterns seen in mammals and birds [33]. The Z chromosome, too, lacks substantial heterogeneity over larger spatial scales, which perhaps reflects the uniform distribution of repetitive elements along its length [24]. Only the autosomal microchromosomes of *P. vitticeps* bear any similarity in GC distribution to the other sauropsid genomes examined. The *P. vitticeps* genome, therefore, has compositional patterns distinct from that of *A. carolinensis*, which indicates that different processes have shaped the genomes of the two lizards since they shared a common ancestor 144 million years ago.

### Comparison with other assemblies

*P. vitticeps* and *A. carolinensis* had similar scaffold N50 values (2.29 Mbp and 4.03 Mbp, respectively). These values for *P. vitticeps* are surprisingly good, as its genome was assembled from short read sequences, whereas that of *A. carolinensis* was generated using Sanger sequencing. Our assembly compares well to nine other sauropsid genomes, including those of two squamates, two turtles and three crocodilians (Table 8).

**Table 8:** Comparison of sequencing platform, assembler, and assembly statistics for the reptiles for which a genome sequence is available

	Bearded dragon	Burmese python	King cobra	Saltwater crocodile	Chicken	Green anole	American alligator	Gharial	Chinese softshell turtle	Green sea turtle	Western painted turtle
	*Pogona vitticeps*	*Python bivittatus*	*Ophiophagus hannah*	*Crocodylus porosus*	*Gallus gallus*	*Anolis carolinensis*	*Alligator mississippiensis*	*Gavialis gangeticus*	*Pelodiscus sinensis*	*Chelonia mydas*	*Chrysemys picta*
Assembler	SOAP deNovo	SOAP deNovo	CLC NGS Cell (version 2011)	AllPaths (version R41313)	Celera Assembler (version 5.4)	Arachne (version 3.0.0)	Allpaths (version R41313)^a^	SOAP deNovo	SOAP deNovo	SOAP deNovo	Newbler
Sequence method	Illumina HiSeq 2000	Illumina GAIIx & HiSeq 2000, Roche 454	Illumina HiSeq	Illumina GAII & HiSeq 2000	Sanger, Roche 454	Sanger	Illumina GAII & HiSeq 2000	Illumina GAII	Illumina HiSeq 2000	Illumina HiSeq 2000	Roche 454, Illumina, Sanger
Average read depth	85.5X	20X	28X	74X	12X	7.1X	68X	109X	105.6X	110X	15X
Genome size (Gbp)	1.77	1.44	1.36–1.59	2.12	1.20		2.17	2.88	2.21	2.24	2.6
Total bases in contigs (excluding unknown bases, Ns)	1,747,541,145	1,384,532,810	1,380,486,984	2,088,185,434	1,032,841,023	1,701,336,547	2,129,643,287	2,198,585,703	2,106,622,020	2,110,365,500	2,173,204,098
Total bases in scaffolds	1,816,115,349	1,435,035,089	1.66 Gbp	2,120,573,303	1,046,932,099	1,799,143,587	2,174,259,888	2,270,567,745	2,202,483,752	2,208,410,377	2,365,766,571
No. of scaffolds (>100 bp)	543,500	39,113	-	23,365	16,847	6,645	14,645	9,317	19,904	140,023	78,631
N50 scaffold (kbp)	2,291	214	226	204	12,877	4,033	509	2,188	3,351	3,864	6,606
No. of contigs (>100 bp)	636,524	274,244	816,633	112,407	27,041	41,986	114,159	177,282	205,380	274,367	262,326
N50 contig (kbp)	31.2	10.7	5.2	32.7	279	79.9	36	23.4	22.0	29.2	21.3
Repeat content	39.5	31.8	35.2	37.5	9.4	34.4	37.7	37.6	42.47	37.35	9.82
No. protein-coding genes	19,406	17262	-	13,321	15,508	17,472	23,323	14,043	19,327	19,633	--

Information is taken from the NCBI database (http://www.ncbi.nlm.nih.gov/assembly), with additional data from the primary papers in which the findings were originally published. ^a^Manual scaffolding

**Fig. 5:**
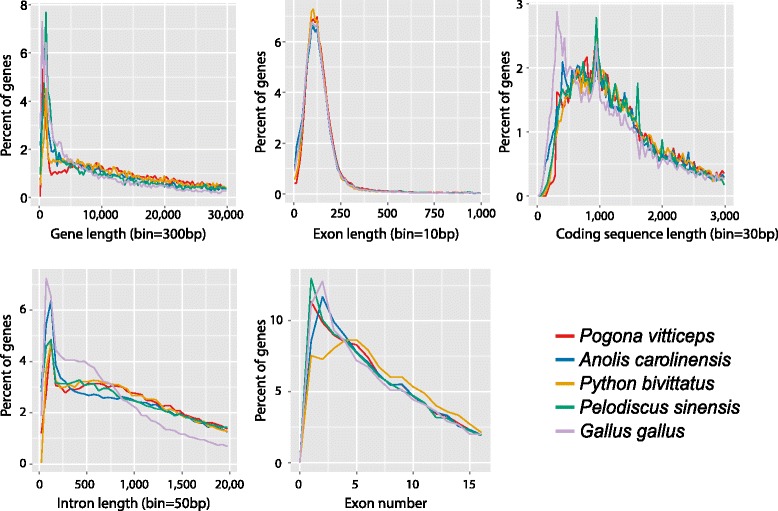
Comparisons of gene parameters among *Pogona vitticeps*, *Gallus gallus*, *Python bivittatus*, *Anolis carolinensis*, and *Pelodiscus sinensis* genomes

The gene parameters listed in Table 6 compare well to those of other vertebrates (see also Fig. 5).

### Concluding remarks

The quality of the *P. vitticeps* assembly is comparable to that of other published squamate genomes. This genome assembly, coupled with the availability of a BAC library and the development of a high-density physical map for each chromosome, provides an unparalleled resource for accelerating research on sex determination, major histocompatibility complex evolution, and the evolution of adaptive traits in squamates to complement the advances brought about by the sequencing of the *A. carolinensis* genome [25].

## Availability of supporting data

The genomic and transcriptomic sequence reads and assemblies have been deposited in the ENA under the project accession number PRJEB5206 (see Additional file 1 for a complete list of accession numbers). The genome sequence has been submitted to GigaDB [34] along with other supporting resources, including:

- SoapDeNovo2 pvi1.1.Jan2013 genome assembly (ENA accession number ERZ094017)
- Trinity *de novo* transcriptome assembly (ENA accession number ERZ097159)
- Peptide and coding sequences for the pvi1.1.Jan2013 assembly
- Gene annotations and repeat annotations for the scaffolds
- Sequence Read Archive accession numbers for all sequencing runs.

The annotated *P. vitticeps* genome sequence can be accessed through a publicly available genome browser [35].

## Additional file

Additional file 1:
**ENA accession numbers.** (XLSX 18 kb)

## Abbreviations

BAC: Bacterial artificial chromosome
bp: Base pair
ENA: European Nucleotide Archive
indel: Sequence insertion or deletion
k-mer: Short sequence of length k
LINE: Long interspersed nuclear element
LTR: Long terminal repeat
N50: 50 % of the genome sequence is contained in contigs (or scaffolds) equal to or greater than this length
ORF: Open reading frame
SINE: Short interspersed nuclear element
SNP: Single nucleotide polymorphism
